# A Cross-Sectional Observational Study of Serum Thyroid-Stimulating Hormone (TSH) in Rheumatoid Arthritis Patients in a Tertiary Care Hospital

**DOI:** 10.7759/cureus.114375

**Published:** 2026-08-11

**Authors:** Yatri Patel, Rahul Patel, Yash K Patel

**Affiliations:** 1 Internal Medicine, Government Medical College, Surat, Surat, IND; 2 Orthopedics and Traumatology, New Civil Hospital, Surat, IND; 3 Medicine, Baroda Medical College, Vadodara, IND

**Keywords:** autoimmune disease, eular, hypothyroidism, rheumatoid arthritis, thyroid dysfunction, thyroid-stimulating hormones

## Abstract

Background: Rheumatoid arthritis (RA) is a systemic autoimmune disease frequently accompanied by thyroid dysfunction, which may influence disease activity. Occasionally, patients with RA have thyroid abnormalities. Autoimmune thyroid conditions, such as Graves’ disease and Hashimoto’s thyroiditis, may have a genetic propensity to occur alongside RA. This study addresses a critical knowledge gap by providing data on the prevalence and patterns of thyroid dysfunction in RA patients in South Gujarat.

Methods: This cross-sectional observational study was conducted at Government Medical College, Surat and the New Civil Hospital, a tertiary care centre in South Gujarat, India, from April 2024 to May 2025, to evaluate the association between serum thyroid-stimulating hormone (TSH) levels and RA severity. Seventy adult patients diagnosed using the European Alliance of Associations for Rheumatology (EULAR) criteria were enrolled through purposive sampling. Demographic, clinical, and laboratory data, including erythrocyte sedimentation rate (ESR), C-reactive protein (CRP), rheumatoid arthritis (RA) factor, anti-cyclic citrullinated peptide (CCP), and thyroid function tests, were collected. Disease activity was assessed via the EULAR criteria.

Results: The cohort was predominantly female (94.29%), with most participants over 40 years of age. Elevated TSH levels were observed in 29.9% of patients, with 15% showing moderate-to-severe hypothyroidism (>10 mIU/L). Significant associations were found between TSH levels and EULAR disease activity scores (χ² = 13.730, p = 0.03). Regression analysis demonstrated that higher TSH (β = 0.312, p = 0.012) independently predicted increased RA severity, whereas the RA factor had no significant impact (p = 0.746). Correlation analysis confirmed strong inverse relationships between TSH and fT4 (r = -0.626, p < 0.001).

Conclusion: These findings indicate that thyroid dysfunction, particularly hypothyroidism, is common in RA and may exacerbate disease severity. Routine thyroid function screening should be considered to optimize management and improve patient outcomes.

## Introduction

In rheumatoid arthritis (RA), a systemic autoimmune disease, chronic synovial inflammation leads to joint destruction, which ultimately causes arthritis [1]. RA is a chronic, debilitating condition with articular and extra-articular manifestations. It affects more women than men, usually between 30 and 50 years of age, while 0.5-1% of the world’s population suffers from it [2,3,4,5,6]. The etiology of RA involves multiple factors, such as genetic, environmental, and immunological components and their complex interplay [7,8,9]. In addition to affecting the musculoskeletal system, RA has various extra-articular manifestations, including cardiovascular, pulmonary, and endocrine complications [10]. Thyroid dysfunction, among them, is of remarkable concern because of its significant implications for disease progression and quality of life.

Thyroid dysfunction is a common comorbidity, with existing literature reporting a prevalence of 15-30% in RA patients. Autoimmune thyroid conditions, such as Graves' disease and Hashimoto's thyroiditis, share a genetic propensity with RA, as per some previous studies [11]. Approximately 6-34% of RA patients have thyroid dysfunction, which varies with demographic region [12]. Hypothyroidism, being the most common ailment, often manifests as subclinical hypothyroidism with normal free thyroxine (T4) levels but increased serum thyroid-stimulating hormone (TSH) levels [13].

The same individual often has multiple coexisting autoimmune disorders owing to shared genetic and environmental risk factors [11]. This phenomenon is present in both RA and autoimmune thyroid disorders, as both exhibit dysregulated immune responses and chronic inflammation, as suggested by increased levels of inflammatory markers, such as C-reactive protein (CRP) and an increased erythrocyte sedimentation rate (ESR) [11]. Their coexistence has been attributed to the presence of autoantibodies, such as rheumatoid factor (RF) and anti-cyclic citrullinated peptide (anti-CCP) in RA, and antithyroid peroxidase (anti-TPO) and antithyroglobulin antibodies in thyroid disorders. Rather than being disease-specific markers, these autoantibodies may depict underlying immune dysregulation [12].

The clinical significance of thyroid dysfunction in RA extends beyond its potential contribution to systemic inflammation [14]. There is an increased risk of cardiovascular complications - a major cause of morbidity and mortality in RA - when there is coexisting subclinical hypothyroidism [15]. Moreover, the efficacy of RA therapies, including disease-modifying antirheumatic drugs (DMARDs) and biologics, may be impaired in patients with untreated thyroid dysfunction. This may be due to altered drug metabolism and immune modulation [16].

The need for integrated care approaches to address the rheumatologic and endocrine aspects of patients’ health has been emphasized by several studies [14,17,18,19]. Furthermore, population-specific investigations are needed to address the regional variations in the prevalence and clinical presentation of thyroid dysfunction. This study addresses a critical knowledge gap by evaluating the prevalence and patterns of thyroid dysfunction in RA patients in south Gujarat and assessing its association with disease activity.

The primary objective was to quantify serum TSH, fT4, and fT3 levels in RA patients diagnosed via American College of Rheumatology (ACR)/European Alliance of Associations for Rheumatology (EULAR) criteria and determine whether thyroid dysfunction independently correlates with disease severity (EULAR criteria score). The need for this study stems from known regional variations in thyroid disorders and the necessity of identifying endocrine comorbidities that may exacerbate rheumatologic outcomes.

## Materials and methods

Study design

This was a cross-sectional observational study carried out at the Department of General Medicine of Government Medical College, Surat and the New Civil Hospital, Surat, Gujarat, India. Seventy patients were selected for the study by conventional purposive sampling. Data collection was carried out from April 2024 to January 2025. The total study period, including data analysis and writing, extended from April 2024 to May 2025. This study was reported in accordance with the Strengthening the Reporting of Observational Studies in Epidemiology (STROBE) guidelines.

Study population

The criteria used for enrolment were as follows:

Inclusion Criteria

Patients diagnosed with RA based on the ACR/EULAR classification criteria [20,21], age ≥18 years (both male and female patients), and patients who provided written informed consent to participate in the study were included.

Exclusion Criteria

This study excluded patients currently on medications known to affect thyroid function (e.g., amiodarone, corticosteroids, and lithium); patients with an ongoing acute or chronic infection, which may influence thyroid function test results; patients diagnosed with malignant thyroid lesions or any known thyroid malignancy; patients who have undergone thyroidectomy (partial or total), as their thyroid function is already altered; patients with chronic liver disease or chronic kidney disease (CKD), as these conditions can impact thyroid hormone metabolism; and patients diagnosed with other autoimmune diseases (e.g., systemic lupus erythematosus, Sjögren’s syndrome, Hashimoto’s thyroiditis, and Graves’ disease), which may independently affect thyroid function.

Data collection

Patient Recruitment

Eligible RA patients visiting the Rheumatology OPD or admitted to the hospital were screened on the basis of the inclusion/exclusion criteria.

After obtaining written informed consent, demographic and clinical details were collected.

Clinical Examination and Data Collection

A structured proforma was used to collect demographic details (age, sex, BMI, etc.), duration of RA and any prior treatments, presence of RA-related complications, and disease activity assessment using the EULAR criteria.

Laboratory Investigations

Venous blood samples (5 mL) were collected from all participants under aseptic precautions. Serum thyroid-stimulating hormone (TSH), free thyroxine (fT4), and free triiodothyronine (fT3) levels were quantitatively measured using chemiluminescence immunoassay (CLIA) on the Roche Cobas e411 automated analyzer (Roche Diagnostics, Basel, Switzerland) in an accredited central laboratory. Erythrocyte sedimentation rate (ESR) was measured using the Westergren method, C-reactive protein (CRP) was quantified via turbidimetric immunoassay, and rheumatoid factor (RF) was determined using latex agglutination/nephelometry.

Statistical analysis

Statistical analysis was conducted using IBM SPSS Statistics for Windows, Version 26.0 (released 2018, IBM Corp., Armonk, NY). A p-value of <0.05 was considered statistically significant. Continuous variables were presented as mean ± standard deviation (SD). Categorical variables were expressed as frequency (%). The chi-square test was used to compare categorical variables. An independent t-test was used to compare continuous variables. Pearson correlation coefficient was used to assess relationships between TSH levels and RA disease activity scores (determined using EULAR criteria). Logistic regression analysis was performed to identify independent predictors of thyroid dysfunction in RA patients, adjusting for potential confounders.

Ethical considerations

The Research Review Committee of Government Medical College, Surat, issued approval (no. 1073/24). Informed written consent was obtained from all the patients or their relatives, permitting the use of their clinical data for research purposes. The procedure for blood sample collection, along with the potential benefits and limitations of the study, was clearly explained to them. To maintain patient confidentiality, personal identifiers, such as name and address, were not recorded in the data spreadsheet. Instead, each patient was assigned a unique identification number, which was used for data entry and analysis. All patient records and study-related documents were securely stored under lock and key, accessible only to the principal investigator. In addition, patients and their relatives were educated about RA, its clinical manifestations, possible complications, and the importance of monitoring thyroid function in managing the disease effectively.

## Results

Demographics

The majority of patients (35.71%) (n = 25) were aged over 50, followed by those aged 41-50 (30%) (n = 21) and 31-40 (28.57%)(n = 20), whereas the youngest group (20-30) accounted for the smallest proportion (5.71%) (n = 4). There was a significant gender disparity, with females comprising the vast majority (94.29%) of patients, whereas males represented a much smaller proportion (5.71%). 

Age group distribution with percentage

The study population comprised 70 patients, predominantly over 40 years of age, with those aged >50 years representing the largest group (35.71%, n = 25), followed by 41-50 years (30.00%, n = 21), 31-40 years (28.57%, n = 20), and 20-30 years (5.71%, n = 4). Females constituted the vast majority of the study population (94.29%, n = 66), while males accounted for 5.71% (n = 4). In terms of occupational background, housewives made up the majority of participants (71.43%, n = 50). The remaining participants were drivers (5.71%, n = 4), teachers (5.71%, n = 4), farmers (4.29%, n = 3), cart-pullers (2.86%, n = 2), shopkeepers (2.86%, n = 2), and maids (2.86%, n = 2).

Symptoms at presentation

Among the presenting complaints, joint pain was almost universally reported, affecting 98.57% of patients (n = 69). Other prominent clinical features included anorexia in 81.43% (n = 57), weakness in 78.57% (n = 55), and morning stiffness in 77.14% of cases (n = 54). Joint swelling was observed in 40.00% of the participants (n = 28). Less common presenting features included menstrual irregularity in 4.29% (n = 3) and weight gain in 1.43% (n = 1).

Medication status for rheumatoid arthritis

The vast majority of patients (94.29%, n = 66) were already on medication, indicating that most cases were not new diagnoses but rather follow-ups or chronic disease management (Table 1). Only a small fraction (5.71%, n = 4) was newly diagnosed.

**Table 1:** Laboratory parameters Values are expressed as mean ± standard deviation (SD) along with the minimum–maximum range. Abbreviations: Hb: hemoglobin; WBC: white blood cell count; ESR: erythrocyte sedimentation rate; CRP: C-reactive protein; RA factor: rheumatoid factor; S.TSH: serum thyroid-stimulating hormone; fT4: free thyroxine; fT3: free triiodothyronine; EULAR: European Alliance of Associations for Rheumatology

Lab Parameter	Mean ± SD	Range
Hb (g/dL)	9.65 ± 2.56	3.0–12.8
WBC (cells/μL)	7967.14 ± 3138.44	2500–16700
ESR (mm/hr)	62.10 ± 38.61	12–140
CRP (mg/L)	41.00 ± 33.77	4–122
RA Factor (IU/mL)	209.04 ± 309.65	23.0–2048.0
S. TSH (mIU/L)	9.17 ± 21.29	0.64–150.10
fT4 (μg/dL)	6.52 ± 2.31	1.12–10.10
fT3 (ng/dL)	104.05 ± 27.06	65.80–168.90
EULAR Criteria (score)	7.79 ± 0.78	7–10

Regarding treatment status at presentation, the vast majority of patients (94.29%, n = 66) were already receiving medication for RA, representing follow-up care or chronic disease management. Only a small minority of participants (5.71%, n = 4) were newly diagnosed and treatment-naïve at the time of enrollment.

EULAR criteria

The majority of patients (84.3%, n = 59) scored a 7 or an 8 on the EULAR criteria, while a smaller proportion (12.9%, n = 9) scored a 9. Only 2.9% (n = 2) reached the maximum score of 10, reflecting very high disease severity.

Evaluation of disease severity using the EULAR criteria revealed that the majority of patients exhibited moderate-to-high disease activity, with 44.3% (n = 31) scoring an 8 and 40.0% (n = 28) scoring a 7. High disease severity was observed in the remaining population, with 12.9% (n = 9) achieving a score of 9 and 2.9% (n = 2) reaching the maximum score of 10, as described further in Table 2.

**Table 2:** Multivariate linear regression analysis of predictors for RA severity (EULAR criteria score) Model evaluated with the EULAR criteria score as the dependent variable (N = 70). * Statistically significant (p < 0.05). Abbreviations: B: unstandardized regression coefficient; Std. error: standard error of the coefficient; beta: standardized regression coefficient; t-value: test statistic; p-value: significance level; RA factor: rheumatoid factor; S.TSH: serum thyroid-stimulating hormone; fT4: free thyroxine; fT3: free triiodothyronine

Predictor	B	Std. error	Standardized β	t-value	p-value
Constant	7.852	0.496	–	15.830	<0.001
RA factor	0.000	0.000	-0.040	-0.325	0.746
S. TSH	0.008	0.003	0.312	2.587	0.012*
fT4	-0.160	0.070	-0.413	-2.284	0.026*
fT3	0.010	0.004	0.353	2.364	0.021*

RA factor and TSH among patients

Nearly half of the patients (48.6%, n = 35) had moderately elevated RA factor levels (101-200 IU/mL), while 24.3%(n = 17) fell in the 51-100 IU/mL range. A notable subset (17.1%, n = 6) exhibited high levels (>200 IU/mL). Ten percent (n = 10) of patients had borderline/low-positive levels (20-50 IU/mL). The majority of patients (70.1%, n = 47) had normal TSH levels (<5.0 mIU/L), while 14.9% (n = 10) showed mild elevation (5.1-10.0 mIU/L). A smaller proportion (15%, n = 13) exhibited moderate to severely elevated TSH (>10.0 mIU/L). Notably, 9% (n = 6) had extremely high TSH (>100 mIU/L). No patient had TSH in the hyperthyroidism range.

Association between the EULAR criteria and RA factor

The chi-square analysis revealed a statistically significant association between RA factor levels and EULAR criteria scores (χ² = 12.115, p = 0.04). Patients with higher RA factor levels (101-200 IU/mL) were most frequently classified under EULAR 7 (40%, n = 28) and 8 (44.3%,n = 31). Notably, no patients with EULAR 9 or 10 (high disease activity) had RA factor >200 IU/mL.

Association between the EULAR criteria and serum TSH

The chi-square test showed a significant association between serum TSH levels and EULAR disease activity scores (χ² = 13.730, p = 0.03). Most patients with normal TSH (<5.0 mIU/L) fell under EULAR 7 (67%, n = 47) and 8 (69%, n = 48), indicating moderate disease activity. Interestingly, higher TSH levels (5.1-20.0 mIU/L) were also observed in these groups, while severely elevated TSH (>100 mIU/L) was rare and not linked to the highest EULAR scores (9-10).

Association between RA factor and serum TSH

The chi-square analysis revealed a statistically significant association between serum TSH levels and RA factor categories (χ² = 31.03, p = 0.013). Patients with mildly elevated TSH (5.1-10.0 mIU/L) were primarily observed in the 101-200 IU/mL RA factor group, while those with normal TSH (<5.0 mIU/L) were distributed across all RA factor levels. Notably, severely elevated TSH (100-150 mIU/L) occurred only in patients with higher RA factor levels (101-200 IU/mL and >400 IU/mL). However, the absence of severe TSH elevation in lower RA factor groups (20-50, 51-100 IU/mL) implies that thyroid dysfunction may not uniformly correlate with RA severity.

Correlation analysis of clinical parameters

The analysis reveals significant positive correlations between hypothyroidism (TSH) and RA severity markers, with TSH showing strong inverse relationships with fT4 (r = -0.626, p < 0.001) and fT3 (r = -0.339, p = 0.005). While TSH's direct correlation with EULAR scores was nonsignificant (r = 0.014, p = 0.909), its strong interrelationships with thyroid hormones suggest thyroid dysfunction may indirectly influence RA progression. Significant negative correlations were observed between inflammatory markers (ESR/CRP) and EULAR scores (p = 0.011 and p = 0.003, respectively). While the simple linear correlation between TSH and EULAR score was not statistically significant (r = 0.014, p = 0.909), an independent association was established in multivariate regression analysis in Table 3.

**Table 3:** Bivariate correlation matrix of inflammatory markers, thyroid parameters , and EULAR criteria scores (N =70) Values represent Pearson correlation coefficients (r) with corresponding p-values shown in parentheses. * Correlation is statistically significant at the 0.05 level (two-tailed). ** Correlation is statistically significant at the 0.01 level (two-tailed). Abbreviations: ESR: erythrocyte sedimentation rate; CRP: C-reactive protein; RA factor: rheumatoid factor; S.TSH: serum thyroid-stimulating hormone; fT4: free thyroxine; fT3: free triiodothyronine; EULAR: European Alliance of Associations for Rheumatology

Variable	ESR	CRP	RA Factor	S. TSH	fT4	fT3	EULAR
ESR (r)	1	0.343**	0.181	0.090	-0.043	0.036	-0.301*
p-value	NA	0.004	0.134	0.467	0.723	0.768	0.011
CRP (r)	0.343**	1	-0.124	0.010	-0.043	-0.074	-0.350**
p-value	0.004	NA	0.306	0.936	0.724	0.545	0.003
RA Factor (r)	0.181	-0.124	1	-0.050	-0.200	-0.102	-0.041
p-value	0.134	0.306	NA	0.685	0.096	0.403	0.734
S. TSH (r)	0.090	0.010	-0.050	1	-0.626**	-0.339**	0.014
p-value	0.467	0.936	0.685	NA	<0.001	0.005	0.909
fT4 (r)	-0.043	-0.043	-0.200	-0.626**	1	0.571**	-0.143
p-value	0.723	0.724	0.096	<0.001	NA	<0.001	0.239
fT3 (r)	0.036	-0.074	-0.102	-0.339**	0.571**	1	0.145
p-value	0.768	0.545	0.403	0.005	<0.001	NA	0.231
EULAR (r)	-0.301*	-0.350**	-0.041	0.014	-0.143	0.145	1
p-value	0.011	0.003	0.734	0.909	0.239	0.231	NA

Regression analysis of the predictors for the EULAR criteria

The multivariate regression demonstrated that higher TSH levels (β = 0.312, p = 0.012) independently predicted increased RA disease severity (EULAR criteria), despite a non-significant univariate linear correlation (r = 0.014, p = 0.909). Correlation analysis confirmed strong inverse relationships between TSH and fT4 (-0.626, p < 0.001). Higher fT4 levels (β = -0.413, p = 0.026) were associated with lower EULAR disease activity scores, while higher fT3 (β = 0.353, p = 0.021) was linked to higher scores, indicating a complex thyroid-RA relationship. RA factor (p = 0.746) showed no significant impact. Partial regression plots were observed between the EULAR criteria and S.TSH and the EULAR criteria and RA factor.

## Discussion

We carried out an observational study to investigate the relationship between serum TSH levels and RA severity in 70 patients from a tertiary care hospital in South Gujarat. Our study showed a concentration of cases in the 41-50 and >50 age groups [1]. Female patients were predominantly involved (94.29%, n = 66) [6]. This demographic profile aligns with established epidemiological patterns of RA, typically affecting women two to three times more frequently than men, with a peak incidence in the fifth and sixth decades of life [6]. In addition, the occupational distribution revealed that 71.43% (n = 50) of the participants were housewives, correlating with the gender distribution.

The clinical presentation was marked by joint pain (98.57%, n = 69), morning stiffness (77.14%, n = 54), and weakness (78.57%, n = 55), which is in line with the common RA symptoms [3,21]. Moreover, anorexia was frequently reported (81.4%, n = 57) [4]. Forty percent (n = 28) of the patients had joint swelling. These findings suggest not only articular involvement but also systemic involvement in RA [10].

According to laboratory results, a considerable percentage of patients had anaemia (mean Hb = 9.65 ± 2.56 g/dL), with 40% (n = 28) showing pallor on examination. This might be due to chronic illness that RA patients experience [7,8].

Notably, 94.3% (n = 66) of patients were already receiving RA treatment at presentation, which may have influenced inflammatory markers, disease activity measurements, and some counterintuitive correlations observed [9,10].

Laboratory examination showed elevated inflammatory markers, with a mean ESR of 62.10 ± 38.61 mm/hr and CRP of 41.00 ± 33.77 mg/L. We used these values for scoring according to the EULAR standards. Moreover, 12.9% (n = 9) of the patients scored a 9 and 84.3% (n = 59) of patients scored a 7 or an 8. Unexpectedly, significant inverse correlations were observed between inflammatory markers (ESR/CRP) and EULAR scores (p = 0.011 and p = 0.003, respectively), which might be due to treatment effects (as the majority of patients were already on medication) and differences in the timing of blood sampling versus clinical scoring. Therefore, these results should be interpreted cautiously and validated in a longitudinal or treatment-naive study.

In evaluating thyroid status in patients with RA, technical challenges inherent to immunoassays must be considered. Circulating autoantibodies, most notably high-titer RF and heterophile antibodies, can cause significant heterophilic interference in automated two-site sandwich immunoassays (such as CLIA and ECLIA). This cross-reactivity can lead to falsely elevated or falsely suppressed serum TSH values, mimicking true thyroid pathology. A bidirectional link exists between TSH levels and RF: elevated RF titers can spuriously alter measured TSH levels, while underlying thyroid dysfunction can modulate systemic inflammatory pathways and immune complex formation. Currently, a lack of standard solutions, such as routine pre-analytical heterophile blocking tubes or universal dilution protocols in clinical laboratories, presents a diagnostic hurdle. Therefore, unexpected or discordant thyroid hormone panels in active RA cases should prompt secondary validation using alternative assay platforms or blocking reagents to exclude assay artifact before initiating long-term endocrine therapy.

There was a significant variation in the RA factor levels (209.04 ± 309.65 IU/mL), with values between 101 and 200 IU/mL seen in 48.6% (n = 34)of individuals. The wide standard deviation indicates significant heterogeneity in disease activity, with some patients exhibiting markedly high levels suggestive of active RA or other autoimmune conditions [5,12]. These findings support the clinical presentation of joint pain, stiffness, and inflammation, reinforcing the need for further autoimmune profiling and targeted therapy [7]. Although it was noted that extreme RA factor values did not consistently correlate with disease severity, the findings highlight complex heterogeneity in RA progression, where moderate RA factor elevations (101-200 IU/mL) dominate across EULAR categories, but very high levels (>400 IU/mL) were rare even in severe cases. The chi-square analysis showed a statistically significant correlation between RA factor levels and EULAR criteria scores (χ² = 12.115, p = 0.04). In keeping with earlier research demonstrating a discrepancy between serological markers and disease activity, this implied that autoantibody titers might not be a linear predictor of clinical severity.

We could conclude that there was a substantial correlation between the severity of RA and blood TSH levels [11,14]. We found that TSH levels varied significantly (9.17 ± 21.29 mIU/L), with 29.9% (n = 28) exhibiting values above the normal range. In particular, 15% (n = 13) had moderate to severe elevation (>10.0 mIU/L), indicating overt hypothyroidism, and 14.9% (n = 10) had mild elevation (5.1-10.0 mIU/L), indicating subclinical hypothyroidism [13]. The analysis reveals significant positive correlations between hypothyroidism (TSH) and RA severity markers, with TSH showing strong inverse relationships with fT4 (r = -0.626, p < 0.001) and fT3 (r = -0.339, p = 0.005). Notably, no patient had a TSH value consistent with hyperthyroidism.

Although univariate correlation between TSH levels and EULAR criteria was non-significant (r = 0.014, p = 0.909), literature comparison further contextualizes our findings within the broader clinical framework. Our observed serum TSH elevation rate of 29.9% (15.0% overt hypothyroidism and 14.9% subclinical hypothyroidism) in RA patients aligns with previously published data. Specifically, Raterman et al. reported a high prevalence of thyroid dysfunction (up to 27%) in RA and noted that thyroid impairment frequently correlates with poorer clinical management. Furthermore, Elattar et al. demonstrated a 24.0% prevalence of hypothyroidism in RA cohorts, reporting significantly higher inflammatory parameters and disease severity scores in hypothyroid RA patients compared to euthyroid controls. Similarly, Mahagna et al. observed a distinct association between thyroid dysfunction, elevated TSH levels, and higher disease activity. The consistent reporting across these regional and international studies reinforces thyroid dysfunction as a significant comorbidity in RA, directly supporting our finding that elevated serum TSH independently predicts higher disease severity scores (\beta = 0.312, p = 0.012). Chi-square analysis confirmed a statistically significant association between TSH levels and EULAR criteria scores (χ² = 13.730, p = 0.03). The multivariate regression analysis further substantiated this relationship, with higher TSH levels (β = 0.312, p = 0.012) independently predicting increased RA severity after adjusting for potential confounders. These findings demonstrate not only an association but also a dose-dependent relationship between thyroid dysfunction and RA severity.

Our findings are consistent with a correlation between higher EULAR scores and elevated TSH levels. The evidence supporting a clinically meaningful association between hypothyroidism and the severity of RA is strengthened by these investigations.

Evaluating thyroid function in RA introduces significant technical and analytical hurdles due to systemic immune dysregulation. Automated two-site sandwich immunoassays (such as CLIA and ECLIA) rely on specific antibody-antigen binding. However, circulating autoantibodies in RA serum, most notably high-titer RF and endogenous heterophile antibodies, frequently cause severe heterophilic interference.

Mechanism of false results

RF and heterophile antibodies can form a bridge between the capture and signal antibodies in sandwich assays even in the absence of the target antigen, yielding false-positive (spurious elevation) TSH readings. Conversely, steric hindrance or binding competition at key antigenic sites can lead to false-negative (falsely suppressed) TSH results.

Bidirectional Link Between TSH and RF

A distinct bidirectional relationship exists between thyroid hormone regulation and RF expression. High concentrations of serum RF cause artifactual alterations in measured TSH values, while underlying thyroid dysfunction and elevated TSH directly influence autoimmune pathways, potentially enhancing systemic B-cell activation, immune complex deposition, and secondary RF propagation.

Lack of Standardized Solutions

A primary clinical limitation is the lack of universal, pre-analytical mitigation solutions in standard automated laboratories. Routine screening assays rarely incorporate pre-treatment steps such as heterophile blocking tubes (HBT), PEG precipitation, or serial dilution protocols prior to reporting.

Consequently, clinicians evaluating RA cohorts must maintain high vigilance when laboratory TSH values do not correlate with clinical presentation or peripheral thyroid hormone levels (fT4/fT3). Unexpected thyroid hormone panels should undergo secondary validation via alternative assay platforms, serial dilutions, or post-treatment with heterophile blocking reagents to rule out analytical artifacts prior to committing patients to lifelong thyroid hormone replacement.

The correlation analysis revealed significant inverse relationships between TSH and thyroid hormones (fT4: r = -0.626, p < 0.001; fT3: r = -0.339, p = 0.005), confirming the presence of true hypothyroidism rather than isolated TSH elevation. Furthermore, the regression analysis showed that higher fT4 levels (β = -0.413, p = 0.026) were associated with reduced severity, while higher fT3 (β = 0.353, p = 0.021) correlated with worse outcomes. This complex relationship suggests that different thyroid hormone components may influence RA through distinct mechanisms.

## Conclusions

Our cross-sectional observational study reaffirms a significant association between elevated serum TSH levels and increased RA severity in patients from South Gujarat. The findings revealed that 29.9% (n = 21)of RA patients had elevated TSH levels. Multivariate analysis confirmed a positive correlation between TSH values and EULAR criteria scores (β = 0.312, p = 0.012). As this relationship was independent of other clinical variables, the results may suggest that thyroid dysfunction directly influences RA pathophysiology. These results have immediate clinical relevance as they support the recommendation for routine thyroid function assessment in RA patients, specifically for those with inadequate treatment response or unexplained symptom exacerbation. This observational study focused strictly on diagnostic baseline screening parameters (serum TSH and free thyroid hormones) rather than therapeutic trial interventions. By demonstrating a high prevalence of subclinical and overt hypothyroidism, these findings support routine thyroid function screening in RA management. Future interventional studies are warranted to assess whether targeted levothyroxine replacement therapy directly reduces RA disease activity scores. Future researchers shall scrutinize whether treating thyroid dysfunction ameliorates RA disease activity. Moreover, they shall explore the underlying immunological mechanisms that couple these autoimmune conditions by investigating thyroid autoantibodies in relation to RA-specific autoantibodies. Molecular studies shall be done to unravel the exact mechanisms by which thyroid dysfunction influences RA pathogenesis. Besides, an assessment of whether the TSH-RA relationship varies with different RA phenotypes or treatment regimens also warrants an investigation.
